# MicroRNA-423-3p promotes glioma growth by targeting PANX2

**DOI:** 10.3892/ol.2021.12833

**Published:** 2021-06-02

**Authors:** Jing Xu, Jian He, He Huang, Renjun Peng, Jian Xi

Oncol Lett 16: 179-188, 2018; DOI: 10.3892/ol.2018.8636

Subsequently to the publication of this paper, it was drawn to the Editors’ attention by a concerned reader that the western blot data featured in Fig. 3F of the article were strikingly similar to data that had appeared in another article by different authors.

Owing to the fact that the contentious data in the above article had already appeared in another article prior to its submission to *Oncology Letters*, the Editor has decided that this paper should be retracted from the Journal. The authors also requested either a corrigendum or a retraction of this paper on account of problems that has been identified with the antibodies used for the western blotting experiments. Upon consulting the authors, they agreed that the article should be retracted. The Editor apologizes to the readership for any inconvenience caused.

